# Familial focal segmental glomerulosclerosis with Alport-like glomerular basement changes caused by paired box protein 2 gene variant

**DOI:** 10.1007/s13730-023-00830-6

**Published:** 2023-10-28

**Authors:** Yuko Yamada, Hiroki Yokoyama, Ryo Kinoshita, Koichi Kitamoto, Yasuo Kawaba, Shinichi Okada, Takashi Horie, China Nagano, Kandai Nozu, Noriyuki Namba

**Affiliations:** 1 Division of Pediatrics and Perinatology, Faculty of Medicine, Tottori University, 36-1, Nishi-Cho, Yonago, Tottori 683-8504 Japan; 2 Department of Pediatrics, Tottori Prefectural Kousei Hospital, Kurayoshi, Tottori Japan; 3 Department of Pediatrics, Yonago Medical Center, Yonago, Tottori Japan; 4 Laboratory of Electron Microscopy, Tottori University, Yonago, Tottori Japan; 5 Department of Pediatrics, Kobe University Graduate School of Medicine, Kobe, Japan

**Keywords:** *PAX2*, Focal segmental glomerulosclerosis, Glomerular basement membrane, Alport-like

## Abstract

Paired box protein 2 (*PAX2*) gene variant causes renal coloboma syndrome (MIM#120330). Further, they are associated with focal segmental glomerulosclerosis and characterized by basement membrane changes similar to Alport syndrome.

Herein, we report an 8-year-old boy who presented with proteinuria and decreased renal function. His paternal uncle has focal segmental glomerulosclerosis and renal failure, and his paternal grandmother has renal failure and is receiving peritoneal dialysis. Further, his father has stage 2 chronic kidney disease. At 3 years of age, his serum creatinine-estimated glomerular filtration rate was 40–50 mL/min/1.73 m^2^. At 8 years of age, his renal function further decreased and he had proteinuria (urinary protein/Cr 3.39 g/g Cr). Renal histopathology showed oligonephronia and focal segmental glomerulosclerosis. A partial basket-weave pattern, similar to Alport syndrome, was also observed on a transmission electron microscope, and low-vacuum scanning electron microscopy revealed coarse meshwork changes in the glomerular basement membrane. Genetic analysis revealed a *PAX2* heterozygous variant (NM_003987.4:c.959C  >  G), a nonsense variant in which the serine at position 320 changes to a stop codon, in our patient and his father. PAX2 is a transcription factor that is important for the podocyte variant. However, podocytes with *PAX2* gene variants may cause abnormal basement membrane production and repair, thereby resulting in Alport-like changes.

## Introduction

Paired paired box protein 2 (*PAX2*) gene variant causes renal coloboma syndrome (RCS, MIM#120330). The common renal findings of RCS include different congenital anomalies of the kidney urinary tract associated with hypoplasia (65%), vesicoureteral reflux (14%), renal cyst (8%), and multiple dysplastic kidney (6%) [1, 2]. The pathological finding is oligomeganephronia caused by the *PAX2* variant [3]. However, focal segmental glomerular sclerosis (FSGS) is associated with the *PAX2* gene variant, and a previous report has shown that 4% of adult-onset familial FSGS is attributed to the *PAX2* gene variant [4]. By contrast, according to a recent case report, Alport-like glomerular basement membrane (GBM) changes and *PAX2* gene variant on electron microscope [5] indicates an association between *PAX2* gene variants and GBM changes. Herein, we report a male patient with familial FSGS associated with a nonsense variant in the *PAX2* gene based on genetic analysis and Alport-like changes in the GBM on electron microscopy of renal tissues.

## Case report

An 8-year-old boy presented with proteinuria and decreased renal function. He was a preterm baby with low birth weight at 33 weeks 2 days of gestation. The patient’s birth weight was 1768 g, and his neonatal distress Apgar score was 2/5/7 because of partial early placental abruption. Renal dysfunction was observed after birth. Voiding cystourethrography showed no vesicoureteral reflux, and abdominal computed tomography (CT) scan revealed no abnormal renal morphology at the age of 1 year. However, the patient’s serum creatinine-estimated glomerular filtration rate was low (40–50 mL/min/1.73 m^2^) at 3 years of age. Thus, treatment with spherical adsorption carbon and angiotensin receptor blocker was started at the age of 4 years. At 8 years of age, the patient’s height and weight were 126.5 cm (+ 0.2 standard deviations [SD]) and 29.8 kg (+ 0.6 SD). His blood pressure was 110/65 mmHg, and there were no significant physical findings. His blood biochemistry test results were as follows: serum creatinine level, 1.31 mg/dL; cystatin C level, 2.26 mg/dL; and serum creatine and cystatin C estimated glomerular filtration rates, 35.2 and 38.26 mL/min/1.73 m^2^, respectively. The urinary findings revealed a protein/creatinine level of 3.39 g/g  ·  Cr and β2-microglobin level of 28,536 μg/L. Renal ultrasonography showed that the size of the kidneys decreased (right kidney: 6.9  ×  3.2 cm [–2.1 SD]; left kidney, 6.3  ×  4.2 cm [–3.0 SD]). Further, a small cyst was found in the right kidney. Hearing loss and ophthalmologic abnormalities were not observed. Open renal biopsy and genetic testing were performed to evaluate the cause of decreased renal function.

## Family history

The patient’s father had stage 2 chronic kidney disease with occasional proteinuria. The paternal grandmother had renal failure of an unknown cause diagnosed 3 years ago and was receiving peritoneal dialysis. The paternal uncle had developed proteinuria when he was a junior high school student. However, he did not receive medical care and was diagnosed with FSGS in his twenties. Further, he was under chronic renal failure management.

## Renal histology

**Fig. 1:**
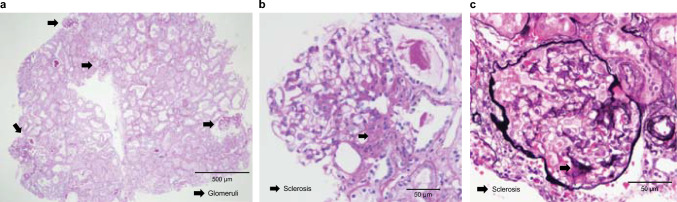
Pathological findings of kidney biopsy under light microscopy. **a** The glomerular number is only five in all tissues collected (1.0/mm^2^). Periodic acid-Schiff staining: original magnification, × 40; scale bar = 500 μm. **b** The glomeruli were hypertrophic, and focal segmental glomerulosclerosis (FSGS) was observed. Periodic acid-Schiff staining: original magnification, × 200; scale bar = 50 μm. **c** Periodic acid–methenamine–silver staining: original magnification, × 200; scale bar = 50 μm

**Fig. 2:**
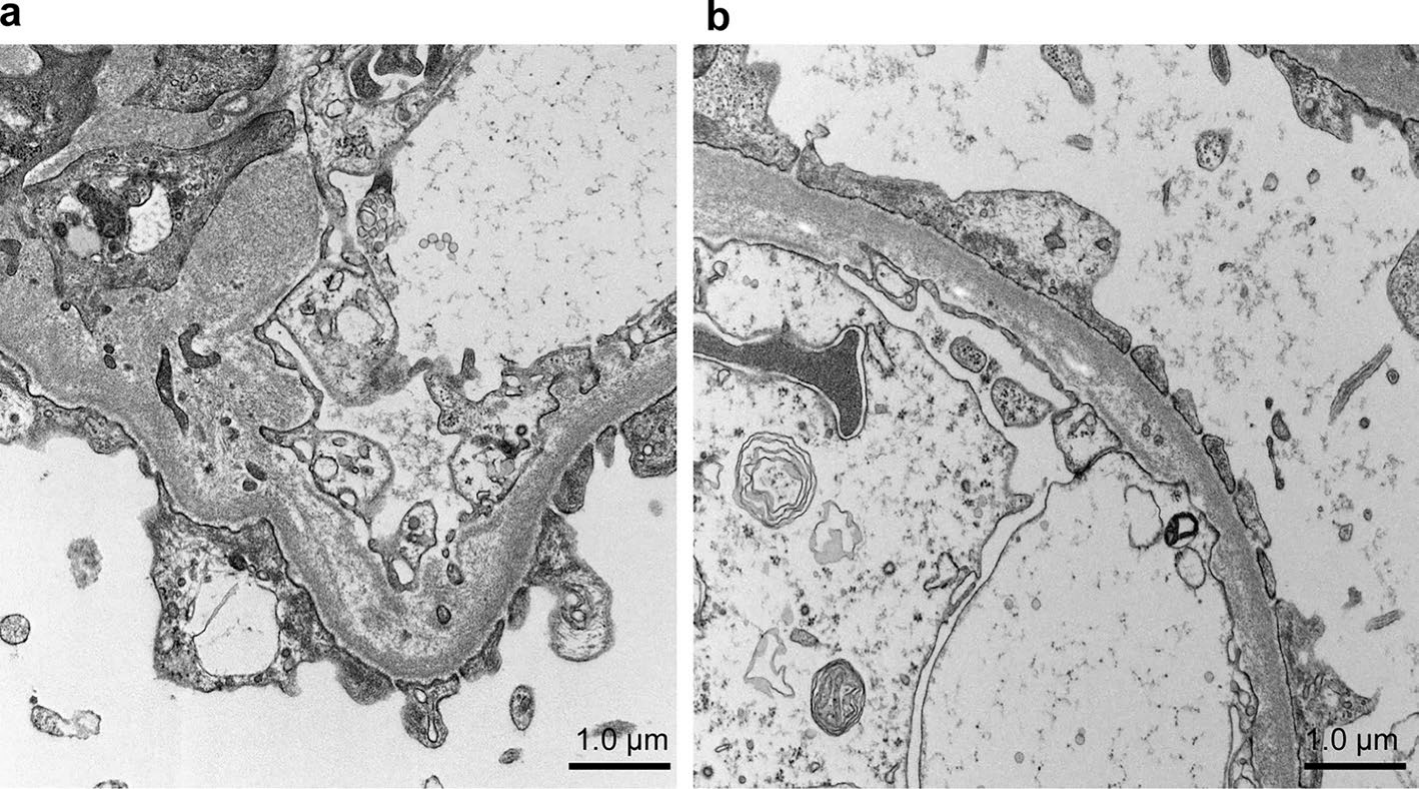
Electron microscopy. **a** The glomerular basement membrane was thick or thinning with a basket-weave pattern, which indicated an Alport syndrome-like change. Scale bar = 1.0 μm. **b** Results showed thickening and thinning of the GBM. Scale bar = 1.0 μm

**Fig. 3:**
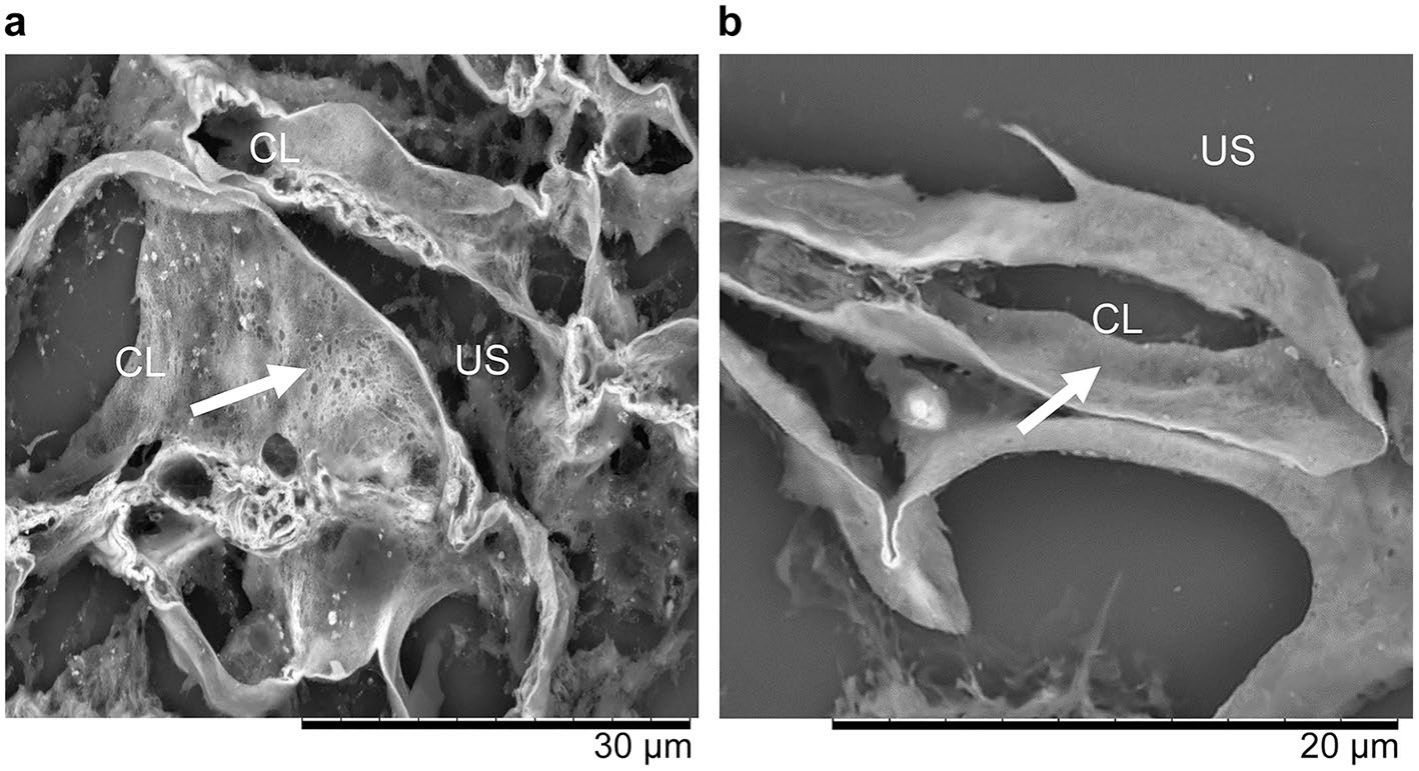
Low-vacuum scanning electron microscopy (LVSEM). **a** Low vacuum scanning electron microscopy (LVSEM) image of the same specimen and glomerulus, as shown in Fig. 1. The glomerular basement membrane (GBM) surface had coarse meshwork structures on the capillary lumen side (arrow) (× 3000). **b** LVSEM image of a normal glomerulus taken from reference 9. The normal GBM appears thin and smooth at the capillary lumen side of the GBM surface (white arrow) (× 5000). *CL* capillary lumen, *US* urinary space

To validate the cause of decreased renal function, an open renal biopsy was performed. A light microscopy specimen was oligonephronic and contained only five glomeruli (approximately 1.0 glomeruli/mm^2^) with diameters ranging from 95 to 222 µm. Some of the glomeruli presented with segmental sclerosis (Fig. 1). Immunofluorescent staining showed negative results. Transmission electron microscopy (TEM) revealed thickening or thinning of the GBM in addition to other findings, such as basket-weave pattern similar to Alport syndrome (AS) (Fig. 2). Low-vacuum scanning electron microscopy (LVSEM) showed no basket-weave in the GBM of any of the five glomeruli, but did show coarse meshwork changes (Fig. 3a). In previous reports, the normal GBM appeared thin and smooth on LVSEM (Fig. 3b) [6]. Moreover, in AS, the basket-weave structure of the GBMs can be observed on LVSEM [7].

## Genetic analysis

**Fig. 4:**
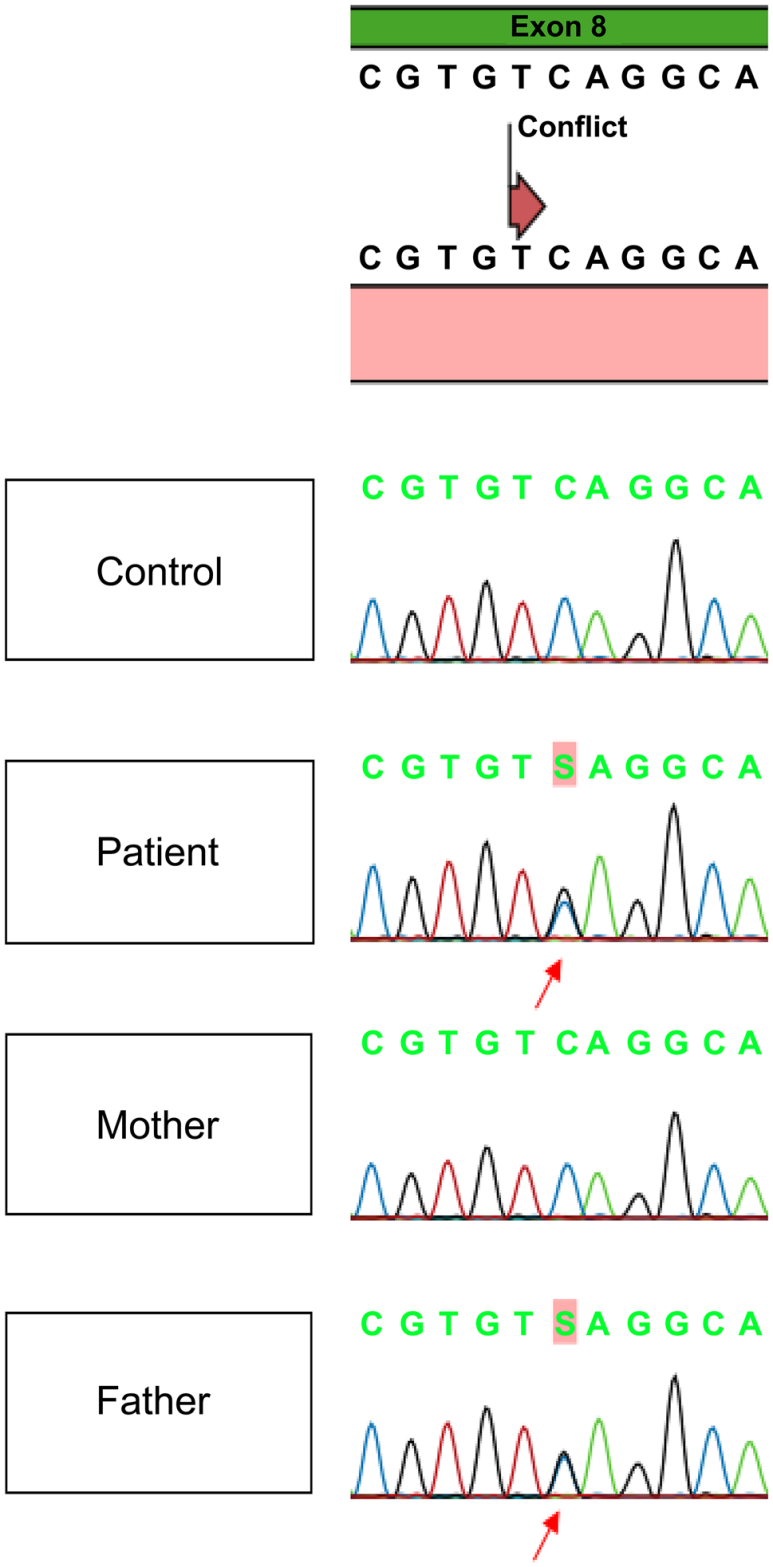
Genetic analysis revealed that both patient and father had *PAX2* variant (c.959C > G)

To validate the cause of decreased renal function, a genetic analysis was performed on our patient and his parents. Heterozygous variant of the *PAX2* gene (NM_003987.4: c.959C  >  G) was detected in one nonsense variant in which the serine at position 320 was changed to a stop codon (Fig. 4). The *PAX2* variant was confirmed in our patient and his father.

## Discussion

We identified a single family with a heterozygous gene variant of the *PAX2* gene in exon 8 (c.959C  >  G). The phenotype of renal symptoms was presumably caused by *PAX2* gene variants. In this case, oligonephronia with few glomeruli was observed on the open renal biopsy tissue. More than half of all glomeruli had a diameter > 200 μm, indicating glomerular hypertrophy. In oligonephronia, the pathogenesis of secondary FSGS is associated with glomerular hypertrophy owing to glomerular hyperfiltration [8]. The risk of oligomeganephronia owing to the *PAX2* variant could not be ruled out in this case. The patient also had been a preterm baby at 33 weeks 2 days of gestation, and his birth weight was 1768 g. Nephron formation in humans begins at 9 weeks and ends at 34–36 weeks of gestation; however, there was no evidence of postnatal nephrogenesis. Perinatal abnormalities, preterm birth, and low birth weight are known risk factors for decreased nephron counts, and infants exposed to low nutrition during this period in utero reportedly have low birth weights and low estimated glomerular filtration rates as adults [9, 10]. The patient had an appropriate birth weight relative to weeks of gestation, and it was unlikely that the patient received poor nutrition before birth. Although it is possible that the preterm birth itself had something to do with the decrease in the number of nephrons, nephron formation is almost complete at 33 weeks of gestation, and it is unlikely that the extreme decrease in the number of glomeruli led to secondary FSGS. The low Apgar score at birth also suggests that renal ischemia owing to circulatory failure immediately after birth may have reduced the number of nephrons. Therefore, the possibility of secondary FSGS due to oligomeganephronia owing to *PAX2* variants or perinatal abnormalities cannot be completely ruled out. However, since the patient had severe proteinuria and there was a family history of FSGS in the paternal side of the family, familial FSGS caused by *PAX2* gene variants was considered.

Further, GBM changes, such as laminar variations, similar to AS, were observed on TEM. In the study of Ohtsubo et al., GBS Alport-like changes were observed in sisters with RCS associated with *PAX2* gene variants in exon 2 (c.76dup, p.Val26Gly fsx27) [5]. The GBM comprises a basic skeleton of type IV collagen, with a combination of laminin, proteoglycans, and entactin are combined. Podocytes are combined by avβ3 integrin to type IV collagen α-chains that comprise the GBM. Podocytes produce and repair the GBM.

PAX2 is a transcription factor expressed in the Wolffian tube and ureteric buds in the embryonic kidney. Among the transcription factors, PAX2 and WT1 have important roles in podocyte maturation [11]. In this case, exon 8 with a heterozygous variant corresponded to the transactivation domain [12]. Therefore, we concluded that podocytes associated with *PAX2* gene exon8 also may have caused abnormal production and repair of basement membranes, resulting in Alport-like changes. However, there have been no previous reports of exon 8 abnormalities in relation to significant changes in the GBM as evaluated using an electron microscope. Further studies are required to clarify GBM changes caused by the *PAX2* variant.

In the current case, LVSEM of the GBM revealed coarse meshwork changes. In the previous reports, the normal GBM exhibited thin and smooth on LVSEM (Fig. 3b) [6]. Moreover, in AS, the basket weave structure of the GBMs can be observed on LVSEM [7]. GBM changes caused by *PAX2* variants may be similar to those in AS on TEM. However, they may present with coarser changes compared with the reticular structure in AS on LVSEM, which provides a three-dimensional structural view. LVSEM can visualize pathophysiological differences. AS is a structural abnormality caused by variants in type IV collagen in the GBM. Meanwhile, the *PAX2* variant is an abnormality of the GBM caused by podocyte irregularities. Hence, more cases should be collected and assessed in the future.

In conclusion, familial FSGS with Alport-like GBM changes caused by the *PAX2* variant was diagnosed in an 8-year-old boy with proteinuria and decreased renal function.
